# Mapping the antimicrobial susceptibility of methicillin-resistant *Staphylococcus aureus* in western Ethiopia: a retrospective cross-sectional analysis

**DOI:** 10.1128/spectrum.00856-26

**Published:** 2026-07-20

**Authors:** Endalu Tesfaye Guteta, Abdi Diriba, Kume Tesfaye, Essa Kedir, Milkias Wakgari, Demeke Jabessa, Motuma Chali, Keresa Biyena, Getu Sileshi, Girmaye Jobir

**Affiliations:** 1 Nekemte Public Health Research and Referral Laboratory Center, Nekemte, Ethiopia; 2 Armaeur Hansen Research Institute, Addis Ababa, Ethiopia; 3 Department of Medical Laboratory Sciences, Institute of Health Sciences, Wollega University 128159, Nekemte, Oromia, Ethiopia; Icahn School of Medicine at Mount Sinai, New York, New York, USA; Debre Markos University, Debre Markos, Ethiopia

**Keywords:** methicillin-resistant *S. aureus*, antimicrobial resistance, multidrug-resistant MRSA

## Abstract

Methicillin-resistant *Staphylococcus aureus* (MRSA) has become a major public health challenge because of its ability to acquire resistance to multiple classes of antibiotics, persist in healthcare environments, and cause severe infections ranging from skin and soft tissue infections to invasive diseases such as sepsis, pneumonia, osteomyelitis, and surgical site infections. Despite its growing clinical significance, there is limited evidence on the long-term trends, antimicrobial resistance patterns, and burden of multidrug-resistant (MDR) MRSA in many resource-limited settings, including Ethiopia. This study aimed to assess the prevalence, temporal trends, and antimicrobial resistance profiles of MRSA isolates over a 5-year period at Nekemte Public Health Research and Referral Laboratory Center. Over the 5 years, 545 *S. aureus* were isolated, among which 67.2% were MRSA. The study revealed significant changes in the distribution of MRSA over 5 years (*P* < 0.001). Of the total MRSA, about 43.2% and 55.5% were isolated from patients aged ≥25 years and male patients, respectively. The majority of the isolates (85.2%) were from hospitals, with outpatient departments being the main source, accounting for 88.5%. Middle ear discharge was the main source of samples for MRSA, which accounted for 67%. Gentamicin showed the highest susceptibility (72.1%), while penicillin resistance was nearly universal (96.7%). Moreover, 93.7% of the MRSA isolates were multidrug resistant, with a high multiple antibiotic resistance index. The prevalence of MDR-MRSA varied significantly across health facilities (*P* < 0.001), patient location (*P* = 0.002), and specimen types (*P* < 0.001).

**IMPORTANCE:**

To address the scarcity of antimicrobial resistance data that undermines patient care, this first multicenter study mapped methicillin-resistant *Staphylococcus aureus* susceptibility in western Ethiopia. The findings guide empiric therapy, establish a stewardship baseline, and contribute to global surveillance. The widespread multidrug resistance and high resistance indices reflect strong antimicrobial pressure and misuse, underscoring the urgent need for antimicrobial stewardship, surveillance, and infection control.

## INTRODUCTION

*Staphylococcus aureus* is a gram-positive, catalase- and coagulase-positive commensal bacterium that colonizes the skin and nares of approximately 30% of individuals (1). It inhabits moist mucous membranes and can cause invasive infections if it enters sterile areas (2). Up to 100% of patients experience transient nasal carriage, with many infections arising from prior colonization (3). *S. aureus* causes serious diseases, including meningitis, septicemia, pneumonia, endocarditis, and osteomyelitis (4, 5), demonstrating exceptional adaptability through antibiotic resistance (6).

Methicillin-resistant *Staphylococcus aureus* (MRSA) is a strain resistant to β-lactam antibiotics (penicillins and cephalosporins), making infections difficult to treat due to the associated multidrug resistance (MDR) (7, 8). Resistance is mediated by the *mecA* gene, which produces an altered penicillin-binding protein (PBP2a) with a reduced affinity for β-lactams (9–11). Epidemiologically, MRSA is classified as community-associated (CA-MRSA), healthcare-associated (HA-MRSA), or livestock-associated (12–18). CA-MRSA is typically susceptible to non-β-lactam antibiotics, whereas HA-MRSA is often multidrug resistant (19).

MRSA is a global health threat linked to 64% higher mortality than non-resistant infections and is listed as a high-priority pathogen by the WHO (20, 21). It is highly prevalent in Europe and the United States and is responsible for >50% of hospital-acquired infections (22). MRSA affects approximately 29% of carriers, causing prolonged hospital stays, increased costs, and thousands of deaths globally, with an estimated 171,000 invasive infections annually in Europe alone (23, 24). Treatment is limited to a few agents (vancomycin and teicoplanin) because of resistance (25). In sub-Saharan Africa, limited surveillance data show MRSA prevalence ranging from 1.25% to 46% (26), while in East Africa, it is 53.4% (27). In Ethiopia, the prevalence varies widely: 68.5% in Gondar (28), 36.9% in Debre Markos (29), 7.5% in Addis Ababa (30), 11.2% in Harar (31), and 17.9% in Hawassa (32). Due to the limited number of short-term studies, this 5-year assessment (2021–2025) examined the prevalence of MRSA and its antimicrobial susceptibility in western Ethiopia.

## MATERIALS AND METHODS

### Study area, design, and period

This laboratory-based analysis was conducted at the Nekemte Public Health Research and Referral Laboratory Center, a regional laboratory in the East Wallaga Zone of the Oromia Region. This laboratory serves more than 13 million people in the catchment, including nearby regions. The center provides diagnostic, research, and mentorship services to catchment health facilities and serves as an antimicrobial resistance surveillance site in western Ethiopia. A retrospective cross-sectional study design was employed. Data of patients referred to Nekemte Public Health Research and Referral Laboratory Center from nearby health facilities between 1 January 2021 and 31 December 2025 were analyzed.

### Data collection

All clinical specimens positive for *S. aureus* were extracted. Patient demographic characteristics (age and sex), type of referring health facilities (hospitals, health centers, and clinics), patient location (outpatient or inpatient), specimen type, and antimicrobial susceptibility profiles were extracted from the laboratory registers.

### Clinical specimen processing, *S. aureus* isolation, and identification

Clinical specimens, including ear discharge, blood, urine, pus, body fluids, sputum, and nasal swabs, from healthcare facilities meeting the acceptance criteria were cultured on blood and mannitol salt agar at 37°C for 24 h. Presumptive *S. aureus*, identified by golden-yellow colonies on mannitol salt agar and beta-hemolysis on blood agar, was confirmed via Gram staining, catalase, and coagulase tests.

### Operational definitions

#### Methicillin-resistant *S. aureus*

*S. aureus* isolates with a cefoxitin zone diameter ≤21 mm on Mueller–Hinton agar after 16-18 h of incubation (29).

#### Multidrug resistance

Non-susceptibility to at least one antimicrobial agent in three or more distinct antimicrobial classes (33).

#### Multiple antibiotic resistance index

Calculated for each isolate by dividing the number of antibiotics to which the isolate was resistant by the total number of antibiotics tested (34–36).

### Antimicrobial susceptibility testing

Methicillin resistance was determined using the cefoxitin (30 µg) disk diffusion method according to CLSI guidelines, with zone diameters ≥22 mm interpreted as susceptible and ≤21 mm as MRSA. MRSA isolates were tested on Mueller–Hinton agar using a 0.5 McFarland suspension and incubated at 37°C for 24 h. The antibiotics tested included penicillin G (10 IU), cefoxitin (30 µg), clindamycin (2 µg), cotrimoxazole (25 µg), azithromycin (15 µg), gentamicin (10 µg), ciprofloxacin (5 µg), chloramphenicol (30 µg), and tetracycline (30 µg).

### Quality assurance

All culture media and antimicrobial disks were routinely quality checked using ATCC reference strains (*Escherichia coli* 25922, *Pseudomonas aeruginosa* 27853, and *S. aureus* 25923) to ensure accurate and reliable testing.

### Data analysis and interpretation

Data quality was checked by using the standard reporting feature; records with missing information were not included in the analysis. Sociodemographic characteristics and antimicrobial susceptibility data were entered into EpiData V4.6 and exported to SPSS version 27 for analysis. Participant characteristics and bacterial isolate profiles were summarized using descriptive statistics. Antimicrobial susceptibility patterns were determined by calculating the percentage of isolates that were classified as resistant, intermediate, or susceptible to each antimicrobial agent tested. The prevalence of MRSA and MDR isolates was calculated as percentages. Associations between categorical variables were assessed using the chi-square test. Temporal trends in MRSA prevalence across the study years were evaluated using the Cochran–Armitage trend test. Results were presented using tables, frequencies, percentages, and summary statistics. A *P* value of less than 0.05 was considered statistically significant.

## RESULTS

### Sociodemographic characteristics and frequency of MRSA isolation

The patients ranged in age from 1 to 82 years (mean ± SD: 21.5 ± 17.5 years). Of the 2,742 specimens collected, 1,419 (51.8%) yielded bacterial growth. Among these, 545 (38.4%) were *S. aureus*, and 366 (67.2%) were MRSA. Of the MRSA isolates, 158 (43.2%) were from patients whose age was ≥25 years. Moreover, 203 (55.5%) isolates were recovered from male patients (Table 1).

**TABLE 1:** Frequency of methicillin-resistant and methicillin-susceptible *S. aureus* with respect to patient characteristics, western Ethiopia, 2021–2025^*b*^

Variable	Category	MRSA (%)	MSSA (%)	*χ*²	*P* value
Age	<5	97 (26.5)	42 (23.5)	4.88	0.181
	5–14	47 (12.8)	32 (17.9)		
	15–24	64 (17.5)	39 (21.8)		
	≥25	158 (43.2)	66 (36.9)		
Sex	Male	203 (55.5)	94 (52.5)	0.422	0.516
	Female	163 (44.5)	85 (47.5)		
Type of referring health facility	Hospital	312 (85.2)	156 (87.2)	0.833	0.659
	Health center	21 (5.7)	7 (3.9)		
	Clinic	33 (9.1)	16 (8.9)		
Patient location	Outpatient department	324 (88.5)	159 (88.8)	0.011	0.917
	Inpatient department	42 (11.5)	20 (11.2)		
Type of clinical specimen	Middle ear discharge	245 (67)	129 (72.1)	5.108	0.164
	Blood	32 (8.7)	12 (6.7)		
	Urine	26 (7.1)	5 (2.8)		
	Others^*a*^	63 (17.2)	33 (18.4)		

^*a*^ Includes pus, body fluids, sputum, and nasal swab.

^*b*^ The proportion of MRSA was calculated from 366 specimens, while the proportion of methicillin-susceptible *Staphylococcus aureus* (MSSA) was calculated from 179 specimens.

### Trend of MRSA over the 5 years

Annual MRSA cases rose steadily from 2021 to 2025, increasing from 49 to 60 in 2022, then to 75 in 2023, followed by a modest rise to 80 in 2024, before surging to a peak of 102 in 2025. The proportion of MRSA among *Staphylococcus aureus* isolates varied significantly across the study years. The Cochran–Armitage trend test demonstrated a significant temporal trend in MRSA prevalence across the study period (*χ*² trend = 11.51, *P* < 0.001). Nevertheless, the yearly proportions revealed a fluctuating pattern. The chi-square test confirmed a significant association between year and methicillin resistance status (*χ*² = 24.31, *P* < 0.001) (Table 2).

**TABLE 2:** Annual distribution of MRSA and methicillin-susceptible *Staphylococcus aureus* (MSSA) isolates, western Oromia, Ethiopia, 2021–2025^*a*^

Year	MRSA (%)	MSSA (%)	Total	Pearson chi-square test		Cochran–Armitage trend test	
				Statistic	*P* value	Statistic	*P* value
2021	49 (72.1)	19 (27.9)	68	*χ*² = 24.31	<0.001	*χ*² trend = 11.51	*<*0.001
2022	60 (78.0)	17 (22.0)	77				
2023	75 (82.4)	16 (17.6)	91				
2024	80 (56.7)	61 (43.3)	141				
2025	102 (60.7)	66 (39.3)	168				

^*a*^ Percentages were calculated within each year. The Cochran–Armitage trend test assessed the linear trend in the proportion of MRSA across the study years, while the Pearson chi-square test evaluated the overall association between year and methicillin resistance status. Both tests indicated a statistically significant temporal change in the distribution of MRSA and MSSA isolates during 2021–2025.

### Antimicrobial susceptibility patterns of MRSA

Antimicrobial susceptibility testing of MRSA isolates using the disk diffusion method revealed notable variability across the antibiotics tested. The highest susceptibility was observed for gentamicin (264, 72.1%), followed by chloramphenicol (189, 51.6%) and clindamycin (184, 50.3%). In contrast, many MRSA isolates were resistant to penicillin G (354, 96.7%) and tetracycline (262, 71.6%), highlighting a high level of resistance to commonly used antibiotics (Table 3).

**TABLE 3:** Antimicrobial susceptibility patterns of MRSA, western Ethiopia, 2021–2025

Antibiotics tested	Susceptibility test results		
	Susceptible	Intermediate	Resistant
Gentamicin	264 (72.1)	13 (3.6)	89 (24.3)
Ciprofloxacin	152 (41.5)	21 (5.7)	193 (52.7)
Tetracycline	89 (24.3)	15 (4.1)	262 (71.6)
Trimethoprim-sulfamethoxazole	90 (24.6)	22 (6.0)	254 (68.4)
Chloramphenicol	189 (51.6)	9 (2.5)	168 (45.9)
Azithromycin	106 (29.0)	16 (4.4)	244 (66.6)
Clindamycin	184 (50.3)	34 (9.3)	148 (40.4)
Penicillin G	12 (3.3)	0 (0.0)	354 (96.7)
Cefoxitin	0 (0.0)	0 (0.0)	366 (100)

### Multidrug-resistant MRSA and multiple antibiotic resistance index

In this study, 343 (93.7%) MRSA isolates were MDR; 68 (18.6%) were resistant to seven antibiotics. The multiple antibiotic resistance (MAR) index exceeded 0.2 for all MDR isolates, indicating their origin from high-risk environments with substantial antibiotic exposure (Table 4).

**TABLE 4:** Multidrug resistance and multiple antibiotic resistance index of MRSA isolates, western Ethiopia, 2021–2025

Number of antibiotics which the isolate resisted	Number of antibiotics tested	Number of isolates (%)	MAR index
1	9	3 (0.8)	0.1
2	9	20 (5.5)	0.2
3	9	44 (12.0)	0.3
4	9	40 (10.9)	0.4
5	9	56 (15.3)	0.6
6	9	51 (13.9)	0.7
7	9	68 (18.6)	0.8
8	9	56 (15.3)	0.9
9	9	28 (7.7)	1.0

### Distribution of multidrug-resistant MRSA

According to this study, 150 MDR-MRSA isolates were recovered from patients older than 24 years. However, the distribution of MDR-MRSA was not statistically significant (*P* = 0.552). With respect to sex, MDR-MRSA was predominantly reported among males, accounting for 190 (55.4%); however, MDR-MRSA did not differ significantly between male and female patients (*P* = 0.916). Furthermore, the prevalence of MDR-MRSA varied significantly across healthcare facility types (*P* < 0.001). Two hundred ninety-one MDR-MRSA were isolated from patients who came from hospitals (Table 5).

**TABLE 5:** Distribution of multidrug-resistant MRSA (*n* = 343) among patient characteristics, western Ethiopia, 2021–2025^*c*^

Variable	Category	MDR status		*χ*²	*P* value
		MDR	Not MDR		
Age	<5	90 (26.2)	5 (21.7)	2.100	0.552
	5–14	44 (12.8)	5 (21.7)		
	15–24	59 (17.2)	5 (21.7)		
	≥25	150 (43.8)	8 (34.9)		
Sex	Male	190 (55.4)	13 (56.5)	0.011	0.916
	Female	153 (44.6)	10 (43.5)		
Type of referring health facility	Hospital	291 (84.8)	11 (47.8)	23.768	<0.001^*a*^
	Health center	21 (6.1)	7 (30.4)		
	Clinic	31 (9.1)	5 (21.8)		
Patient location	Outpatient department	301 (87.8)	15 (65.2)	9.283	0.002^*a*^
	Inpatient department	42 (12.2)	8 (34.8)		
Type of clinical specimen	Middle ear discharge	228 (66.5)	6 (26.1)	20.613	<0.001^*a*^
	Blood	32 (9.3)	6 (26.1)		
	Urine	25 (7.3)	6 (26.1)		
	Others^*b*^	58 (16.9)	5 (21.7)		

^*a*^ Statistically significant at *P <* 0.05.

^*b*^ Includes pus, body fluids, sputum, and nasal swab.

^*c*^ The proportion of MDR-MRSA was calculated from 343 specimens, while the proportion of non-MDR-MRSA was calculated from 23 specimens.

## DISCUSSION

This study examined MRSA prevalence, susceptibility patterns, and multidrug resistance over 5 years. The prevalence of MRSA was 67.2% (95% CI, 63.1–71.0), which is higher than that reported in Addis Ababa (27%) (37), Hawassa (17.9%) (32), Harar (11.2%) (31), Nigeria (35.4%) (38), Tanzania (48.5%) (39), Saudi Arabia (52.7%) (40), Pakistan (49%) (41), and Nepal (26.4%) (42). This finding was consistent with Gondar (68.5%) (28) but lower than Arba Minch (82.3%) (43), Somalia (81%) (44), and Pakistan (80%) (45). These variations reflect differences in study design, location, sample size, patient groups, laboratory methods, and antibiotic use, underscoring the global public health impact of this issue.

Trend analysis revealed a statistically significant temporal change in the distribution of MRSA isolates during 2021–2025 (*P* < 0.001). MRSA prevalence showed a fluctuating trend over the study period, increasing from 72.1% in 2021 to 82.4% in 2023, followed by a marked decline to 56.7% in 2024 and a slight increase to 60.7% in 2025. Overall, the trend was characterized by an initial rise, a sharp decrease, and a modest rebound in the final year. Other similar studies reported varying trends of MRSA prevalence (37, 46–48). The variation in MRSA prevalence reported across different studies can be attributed to several factors, including differences in study design, healthcare settings, patient populations, specimen types, antimicrobial prescribing practices, infection prevention and control measures, and laboratory methods used for MRSA detection (49).

Age-wise analysis revealed MRSA prevalence among patients aged ≥25 years, accounting for 43.2%. However, no significant association was observed between age category and MRSA occurrence (*P* = 0.181). Other studies from Arba Minch (18–28 years) (43), Debre Markos (15–30 years) (47), Addis Ababa (25–34 years) (33), and Nigeria (31–45 years) (50) reported varying MRSA prevalence. These differences likely reflect variations in the study populations. On the other hand, MRSA was more common in males (55.5%) but consistent with reports from Gondar (28), Addis Ababa (37), Arba Minch (43), Saudi Arabia (40), and Pakistan (41), although some studies found a female predominance (32, 51). In the present study, the distribution of MRSA did not differ significantly by sex (*P* = 0.515). No single biological factor explains sex differences; the prevalence likely reflects a mix of biological, behavioral, and environmental factors (52, 53).

In addition, 85.2% of MRSA isolates were from hospital patients, but there was no significant difference in the distribution of MRSA among health facilities (*P* = 0.659). This study found that 88.5% of MRSA isolates came from outpatients, aligning with one Indian study (54) but contrasting with reports from Addis Ababa (37), Hawassa (32), Gondar (28), and Tanzania (39), where MRSA was more common among inpatients than outpatients. However, the current study revealed no statistically significant association between patient setting (outpatient versus inpatient) and MRSA occurrence (*P* = 0.917). Most isolates were from middle ear discharge (67%), followed by pus (15.3%), even though the differences across specimen types were not statistically significant (*P* = 0.164). This differs from studies from Ethiopia (33, 37), Nigeria (50%) (50), India (35.7%) (54), and Pakistan (51%) (45), where pus was the main source. Such discrepancies may reflect differences in colonization sites, patient populations, antibiotic use, clinical procedures, or detection methods.

Antibiotic susceptibility testing revealed varying resistance patterns. Sensitivity was highest to gentamicin (72.1%), while 96.7% were resistant to penicillin. These findings are consistent with studies reporting gentamicin sensitivity rates of 95.7% and penicillin resistance rates of 93.3% (31), as well as another study in which 50% were gentamicin-sensitive and all were penicillin-resistant (37). Ciprofloxacin resistance was 52.7%, which is similar to that reported in one study (55.3%) (55) but different from that reported in another (86.4%) (56). Over half of the isolates were resistant to trimethoprim-sulfamethoxazole (68.4%), azithromycin (66.6%), and clindamycin (50.3%). Other studies have shown variable patterns globally (33, 41, 43, 51).

MDR was 93.7% (95% CI, 90.6–95.6), exceeding rates in Northwestern Ethiopia (57.1%) (29) and Nigeria (57.7%) (34) but consistent with that in Nepal (94.1%) (42). Discrepancies in MDR-MRSA distribution stem from geography, setting, period, dominant clones, antibiotic use, and methods used. In the current study, the prevalence of MDR-MRSA varied significantly across healthcare facility types (*P* < 0.001). It was found that 84.8% of MDR-MRSA were recovered among patients from hospitals. Hospitals generally report higher MDR-MRSA rates because they manage severely ill patients, prolonged hospitalizations, invasive procedures, intensive antibiotic exposure, and frequent patient-to-patient transmission (57).

Moreover, MDR-MRSA prevalence differed significantly between outpatient and inpatient populations (*P* = 0.002). It was found that 87.8% of MDR-MRSA were isolated from outpatient departments. This finding is similar to one study from India (54), but study findings from Ethiopia showed contrasting results, indicating that most isolates were from inpatient departments (28, 32, 37). The higher frequency of MDR-MRSA in the current study may be due to the fact that most isolates were recovered from middle ear discharges from patients who came from outpatient departments. The higher occurrence of MDR-MRSA among outpatient department patients may be explained by the increasing spread of CA-MRSA strains and widespread antibiotic exposure in the community. Outpatients are more likely to receive repeated empirical antibiotic treatments for skin and soft tissue infections, respiratory infections, and chronic wounds. CA-MRSA has increasingly acquired resistance to multiple antimicrobial classes, making MDR-MRSA a significant problem among outpatient populations (58, 59).

Furthermore, the present study revealed that the distribution of MDR-MRSA varied significantly by specimen type (*P* < 0.001). Middle ear discharge specimens accounted for the largest proportion of MDR-MRSA isolates (66.5%), indicating a significant association between specimen source and multidrug resistance. This may reflect recurrent infections and repeated antimicrobial exposure among patients with chronic otitis media. Contrary to the current study findings, other studies reported pus/wound as the main source of MDR-MRSA (33, 45). The significant association between specimen type and MDR-MRSA may be explained by differences in infection chronicity and prior antibiotic exposure. Chronic infections, particularly those involving middle ear discharge, often require repeated antimicrobial treatment, which can select for and sustain multidrug-resistant MRSA strains. Thus, specimen type may reflect variations in treatment history and resistance development.

In the current study, the MAR index ranged from 0.1 to 1.0. It was found that all MDR-MRSA yielded MAR index values above 0.2. Values >0.2 indicate high-risk sources under strong antibiotic pressure, posing public health concerns (35, 36), while ≤0.2 suggests low antibiotic use settings (34). In the current study, the high MAR index observed among MRSA isolates suggests that these strains originated from environments with frequent antibiotic use, where repeated exposure to multiple antimicrobial agents may have promoted the development and persistence of multidrug resistance.

### Conclusion and recommendation

In conclusion, the present study reveals a high prevalence of MRSA with a considerable proportion of MDR-MRSA isolates and high MAR indices among clinical samples. These results demonstrate high selective antimicrobial pressure and wide diffusion of resistance, providing evidence of frequent exposure of MRSA strains to multiple classes of antibiotics. The pattern of resistance reflects the persistent difficulty in dealing with staphylococcal infections in the study setting. Effective antimicrobial stewardship, regular susceptibility testing, and robust infection prevention and control measures are required to avoid further emergence and spread of resistant MRSA strains.

### Limitations

Methicillin resistance was determined using cefoxitin. Minimum inhibitory concentration was not used for confirmation. Moreover, classification of HA-MRSA and CA-MRSA was not possible except for describing that the isolates were from outpatient departments or inpatient departments based on available information. Molecular identification of resistance genes was not performed, so the genetic basis of observed resistance patterns could not be determined. Additionally, patient- or healthcare-related risk factor data were unavailable, preventing analysis of potential resistance predictors. These gaps limit the depth, comprehensiveness, and generalizability of the findings.

## Supplementary Material

Reviewer comments

## Data Availability

All supporting files are uploaded in figshare with DOI 10.6084/m9.figshare.32767500.

## SUPPLEMENTAL MATERIAL

OPEN PEER REVIEW reviewer-comments.pdf An accounting of the reviewer comments and feedback.

ASM does not own the copyrights to Supplemental Material that may be linked to, or accessed through, an article. The authors have granted ASM a non-exclusive, world-wide license to publish the Supplemental Material files. Please contact the corresponding author directly for reuse.
