# Supplementary material for: Mapping the antimicrobial susceptibility of methicillin-resistant Staphylococcus aureus in western Ethiopia: a retrospective cross-sectional analysis
Source: Microbiol Spectr. 2026 Jul 20;14(9):e00856-26. doi: 10.1128/spectrum.00856-26 (PMC13532236; doi:10.1128/spectrum.00856-26)
Supplement: Supplementary file 1 [file reviewer-comments.pdf]

AMERICAN SOCIETY FOR MICROBIOLOGY | Microbiology Spectrum

Mapping the Antimicrobial Susceptibility of Methicillin-Resistant *Staphylococcus aureus* in Western Ethiopia: A retrospective cross-sectional analysis

Endalu Guteta, Abdi Diriba, Kume Tesfaye, Essa Kedir, Milkias Wakgari, Demeke Jabessa, Motuma Chali, Keresa Biyena, Getu Sileshi, and Girmaye Jobir

Corresponding Author(s): Endalu Guteta, Nekemte Public Health Research and Referral Laboratory Center

Review Timeline:

Submission Date:	March 10, 2026
Editorial Decision:	May 28, 2026
Revision Received:	June 8, 2026
Accepted:	June 14, 2026

Editor: Deena Altman

Reviewer(s): Disclosure of reviewer identity is with reference to reviewer comments included in decision letter(s). The following individuals involved in review of your submission have agreed to reveal their identity: Tebelay Dilnessa (Reviewer #1)

Transaction Report:

(Note: With the exception of the correction of typographical or spelling errors that could be a source of ambiguity, letters and reports are not edited. The original formatting of letters and referee reports may not be reflected in this compilation.)

DOI: https://doi.org/10.1128/spectrum.00856-26

Re: Spectrum00856-26 (Mapping the Antimicrobial Susceptibility of Methicillin-Resistant Staphylococcus aureus in Western Ethiopia: A multicenter cross-sectional study)

Dear Mr. Endalu Tesfaye Guteta:

Thank you for the privilege of reviewing your work. Below you will find my comments, instructions from the Spectrum editorial office, and the reviewer comments.

This manuscript will require a Major Revision based on reviewers comments in order to be further considered. There were concerns that the English language usage in the manuscript might make it difficult to properly evaluate the science. The ASM Journals webpage provides links to various language editing services (Writing Your Paper (asm.org)). You may consider using these services when revising your manuscript. The use of these services will have no direct bearing on the editorial decision. ASM has no affiliation with these companies. As a reminder, AI tools may be used only to improve the language, accessibility, or quality of human-generated text. Please disclose the use of any AI services when revising your manuscript.

Please return the manuscript within 60 days; if you cannot complete the modification within this time period, please contact me. If you do not wish to modify the manuscript and prefer to submit it to another journal, notify me immediately so that the manuscript may be formally withdrawn from consideration by Spectrum.

Revision Guidelines
To submit your modified manuscript, log into the submission site at https://spectrum.msubmit.net/cgi-bin/main.plex. Go to Author Tasks and click the appropriate manuscript title to begin. The information you entered when you first submitted the paper will be displayed; update this as necessary. Note the following requirements:

• Upload point-by-point responses to the issues raised by the reviewers in a file named "Response to Reviewers," NOT in your cover letter.
• Upload a compare copy of the manuscript (without figures) as a "Marked-Up Manuscript" file.
• Upload a clean .DOC/.DOCX version of the revised manuscript and remove the previous version.
• Each figure must be uploaded as a separate, editable, high-resolution file (TIFF or EPS preferred), and any multipanel figures must be assembled into one file.
• Any supplemental material intended for posting by ASM should be uploaded with their legends separate from the main manuscript. You can combine all supplemental material into one file (preferred) or split it into a maximum of 10 files with all associated legends included.

For complete guidelines on revision requirements, see our Submission and Review Process webpage. Submission of a paper that does not conform to guidelines may delay acceptance of your manuscript.

Data availability: ASM policy requires that data be available to the public upon online posting of the article, so please verify all links to sequence records, if present, and make sure that each number retrieves the full record of the data. If a new accession number is not linked or a link is broken, provide Spectrum production staff with the correct URL for the record. If the accession numbers for new data are not publicly accessible before the expected online posting of the article, publication may be delayed; please contact production staff (Spectrum@asmusa.org) immediately with the expected release date.

Publication Fees: For information on publication fees and which article types are subject to charges, visit our website. If your manuscript is accepted for publication and any fees apply, you will be contacted separately about payment during the production process; please follow the instructions in that e-mail. Arrangements for payment must be made before your article is published.

ASM Membership: Corresponding authors may join or renew ASM membership to obtain discounts on publication fees. Need to upgrade your membership level? Please contact Customer Service at Service@asmusa.org.

The ASM Journals program strives for constant improvement in our submission and publication process. Please tell us how we can improve your experience by taking this quick Author Survey.

Thank you for submitting your paper to Spectrum.

Sincerely,
Deena Altman
Editor
Microbiology Spectrum

Reviewer #1 (Comments for the Author):

This manuscript addresses an important and timely public health issue-methicillin-resistant Staphylococcus aureus (MRSA) and its antimicrobial susceptibility patterns in Western Ethiopia. The study is valuable due to its multicenter design, large sample size (n= 545 S. aureus isolates), and five-year trend analysis (2021-2025), which provides meaningful insights into resistance dynamics in a resource-limited setting. The findings-particularly the high MRSA prevalence (67.2%), extensive multidrug resistance (93.4%), and high MARI values-are significant and relevant for antimicrobial stewardship and clinical practice. However, the manuscript requires major revision to improve clarity, methodological rigor, data interpretation, and consistency.
Comments
• The study is described as a "multicenter cross-sectional study", but it spans five years (2021-2025). This is more appropriately described as a retrospective longitudinal or repeated cross-sectional study. Clarify whether: data were collected continuously, or separate cross-sectional snapshots were analyzed annually.
• Abstract: Well-structured but: start by 'Background' and contains interpretation beyond results; "Complex interplay..." is vague. Make abstract more data-driven and concise
• Follow an organized structure based on the authors guide.
• The manuscript states that specimens were collected from: 6 hospitals, 3 health centers, 6 clinics. However, there is no description of sampling strategy (consecutive, random, convenience). No explanation of catchment population or representativeness. Add details on: inclusion/exclusion criteria, whether duplicate isolates per patient were excluded and representativeness of the study population.
• Clarify study design (cross-sectional vs longitudinal), and add inferential statistics
• The manuscript classifies MRSA as: Hospital-associated (85.2%). Yet 88.5% are from outpatients. This is contradictory. Clearly define how HA-MRSA vs CA-MRSA was determined. If classification is based on facility rather than epidemiological criteria, clarify this limitation.
• The analysis is purely descriptive. No statistical tests are reported: No comparison between groups (e.g., age, sex). No trend analysis (despite a 5-year dataset). Include: Chi-square or logistic regression (e.g., MRSA vs MSSA predictors), trend analysis (e.g., regression) and confidence intervals consistently for key outcomes
• Some interpretations are speculative without supporting data: "Increased MRSA burden may stem from poor stewardship or infection control". These claims are reasonable but not directly measured. Rephrase to reflect a hypothesis rather than conclusion. Clearly distinguish data-driven findings vs. assumptions.
• The study uses disk diffusion only, which is acceptable. However, no mention of: D-test for inducible clindamycin resistance. MIC confirmation for key drugs (e.g., vancomycin). Mention whether D-test was performed (important for clindamycin interpretation). Clarify absence of MIC testing as a limitation
• Important anti-MRSA agents are missing: Vancomycin, Linezolid, Teicoplanin. Explain why these were not tested (resource limitation?) Discuss implications for treatment recommendations
• MDR definition is appropriate. However: MARI calculation lacks clarity: were all isolates tested against the same number of antibiotics? Clarify denominator consistency. Explain variation (e.g., nitrofurantoin only for urine isolates)
• Some inconsistencies: Nitrofurantoin totals do not match full sample size. Percentages occasionally unclear (denominator not specified). Ensure all percentages clearly state denominators and separate urine-only analysis where appropriate.
• Several sentences are overly long, grammatically inconsistent, and repetitive. Examples: "Patient demographic characteristics (age and sex), type of referring health facilities..." → incomplete sentence; "Clinical specimens... and antimicrobial susceptibility profiles." → fragment. Perform thorough language editing and consider professional proofreading
• Figure 1: Not clearly labeled (axes unclear) and MSSA vs MRSA trend needs better explanation
• Tables: Formatting inconsistencies and some spacing and alignment issues
• Discussion: Generally good comparison with literature. However: too descriptive and needs more critical analysis.
• Resolve HA vs CA inconsistency and improve antimicrobial testing discussion
• Limitations: Important points mentioned, but missing: Lack of molecular typing, clinical outcome data, and antibiotic usage data.
• The conclusion also requires revision.

Reviewer #2 (Comments for the Author):

This study provides valuable baseline data on MRSA prevalence and antimicrobial resistance in a resource-limited region of Ethiopia. The five-year observation period and the multicenter design are commendable. However, several methodological limitations substantially weaken the validity and generalizability of the findings. The lack of molecular confirmation for MRSA, the cross-sectional design without risk-factor analysis, the strong specimen bias (67% from ear discharge), and the omission of key anti-MRSA agents (vancomycin, linezolid, daptomycin) from susceptibility testing are serious shortcomings. Addressing these issues, at minimum by performing mecA PCR and expanding the antibiotic panel, would be necessary to make the conclusions convincing. Without such improvements, the study remains a preliminary descriptive report rather than a robust epidemiological investigation.

Specific comments:

1. Identification of MRSA relied solely on the phenotypic cefoxitin disk diffusion method. No molecular assays (e.g., mecA gene PCR and PVL toxin gene detection) were performed. Consequently, the study cannot distinguish healthcare-associated (HA-MRSA) from community-associated (CA-MRSA) strains, nor can it elucidate the transmission mechanisms of resistance genes. Although the authors acknowledge this limitation in the manuscript, mere acknowledgment is insufficient. At a minimum, detection of one key resistance gene (e.g., mecA) should be performed; otherwise, the manuscript lacks convincing evidence.
2. The cross-sectional design only allows description of associations; it cannot determine the causal direction (e.g., whether hospitalization leads to MRSA infection or infection leads to hospitalization).
3. Key variables such as previous antibiotic use, hospitalization history, underlying diseases, and medical procedures were not collected. Therefore, a multivariate analysis to identify independent risk factors for MRSA infection could not be performed.
4. Although the manuscript reports that multiple specimen types were collected, 67% of MRSA isolates originated from middle ear discharge, while blood, urine, pus, sputum, and other common infection sites together accounted for only 33%. This distribution markedly differs from global patterns where MRSA is mainly isolated from wounds, the respiratory tract, and blood. The bias may reflect preferential submission of specimens from otitis media patients or a high local prevalence of otitis media. Thus, the results cannot be extrapolated to the overall epidemiology of MRSA in this region; interpretations should be restricted to "otitis-media-associated MRSA" or should explicitly note the referral bias.
5. First-line and second-line agents for treating MRSA infections (e.g., vancomycin, linezolid, daptomycin) were not tested. The study relied on agents such as gentamicin (which is relatively toxic and not the preferred choice for MRSA), greatly reducing the clinical value of the findings for guiding therapy.
6. The presentation of the five-year MRSA trend (Figure 1) is inadequate. Currently, only absolute case numbers are described without graphical support or statistical analysis. The authors should provide a bar chart (or line graph) showing both annual absolute counts and, more importantly, the MRSA detection rate (MRSA / total S. aureus isolates × 100%) for each year. A statistical test for trend, such as the Cochran-Armitage test for trend or logistic regression with year as a continuous variable, should be performed and the corresponding P value reported. This would allow readers to assess whether the observed increase is statistically significant or merely due to random variation or changes in sample submission volumes over time.

This study provides valuable baseline data on MRSA prevalence and antimicrobial resistance in a resource-limited region of Ethiopia. The five-year observation period and the multicenter design are commendable. However, several methodological limitations substantially weaken the validity and generalizability of the findings. The lack of molecular confirmation for MRSA, the cross-sectional design without risk-factor analysis, the strong specimen bias (67% from ear discharge), and the omission of key anti-MRSA agents (vancomycin, linezolid, daptomycin) from susceptibility testing are serious shortcomings. Addressing these issues, at minimum by performing mecA PCR and expanding the antibiotic panel, would be necessary to make the conclusions convincing. Without such improvements, the study remains a preliminary descriptive report rather than a robust epidemiological investigation.

Specific comments:

1. Identification of MRSA relied solely on the phenotypic cefoxitin disk diffusion method. No molecular assays (e.g., mecA gene PCR and PVL toxin gene detection) were performed. Consequently, the study cannot distinguish healthcare-associated (HA-MRSA) from community-associated (CA-MRSA) strains, nor can it elucidate the transmission mechanisms of resistance genes. Although the authors acknowledge this limitation in the manuscript, mere acknowledgment is insufficient. At a minimum, detection of one key resistance gene (e.g., mecA) should be performed; otherwise, the manuscript lacks convincing evidence.
2. The cross-sectional design only allows description of associations; it cannot determine the causal direction (e.g., whether hospitalization leads to MRSA infection or infection leads to hospitalization).
3. Key variables such as previous antibiotic use, hospitalization history, underlying diseases, and medical procedures were not collected. Therefore, a multivariate analysis to identify independent risk factors for MRSA infection could not be performed.
4. Although the manuscript reports that multiple specimen types were collected, 67% of MRSA isolates originated from middle ear discharge, while blood, urine, pus, sputum, and other common infection sites together accounted for only 33%. This distribution

markedly differs from global patterns where MRSA is mainly isolated from wounds, the respiratory tract, and blood. The bias may reflect preferential submission of specimens from otitis media patients or a high local prevalence of otitis media. Thus, the results cannot be extrapolated to the overall epidemiology of MRSA in this region; interpretations should be restricted to "otitis-media-associated MRSA" or should explicitly note the referral bias.

5. First-line and second-line agents for treating MRSA infections (e.g., vancomycin, linezolid, daptomycin) were not tested. The study relied on agents such as gentamicin (which is relatively toxic and not the preferred choice for MRSA), greatly reducing the clinical value of the findings for guiding therapy.

6. The presentation of the five-year MRSA trend (Figure 1) is inadequate. Currently, only absolute case numbers are described without graphical support or statistical analysis. The authors should provide a bar chart (or line graph) showing both annual absolute counts and, more importantly, the MRSA detection rate (MRSA / total S. aureus isolates × 100%) for each year. A statistical test for trend, such as the Cochran-Armitage test for trend or logistic regression with year as a continuous variable, should be performed and the corresponding P value reported. This would allow readers to assess whether the observed increase is statistically significant or merely due to random variation or changes in sample submission volumes over time.

Point-by-point response

1. Reviewer 1

SN	Reviewer comments	Authors' response
1	The study is described as a "multicenter cross-sectional study", but it spans five years (2021-2025). This is more appropriately described as a retrospective longitudinal or repeated cross-sectional study. Clarify whether: data were collected continuously, or separate cross-sectional snapshots were analyzed annually.	Modified as "**Mapping the Antimicrobial Susceptibility of Methicillin-Resistant *Staphylococcus aureus* in Western Ethiopia: A retrospective cross-sectional analysis"** in the manuscript. Data were collected cross-sectionally at the end of 2025.
2	**Abstract**: Well-structured but: start by 'Background' and contains interpretation beyond results; "Complex interplay..." is vague. Make abstract more data-driven and concise	Corrected in the manuscript
3	The manuscript states that specimens were collected from: 6 hospitals, 3 health centers, 6 clinics. However, there is no description of sampling strategy (consecutive, random, convenience). No explanation of catchment population or representativeness. Add details on: inclusion/exclusion criteria, whether duplicate isolates per patient were excluded and representativeness of the study population.	Since this is retrospective, data of 5 years were analyzed after 5 years from laboratory registration book. Here, we raised the health facilities because the patients were referred from these facilities over the years (They were the main sources).<br>Corrected in the manuscript.
4	Clarify study design (cross-sectional vs longitudinal), and add inferential statistics	It is purely retrospective cross-sectional because we too five years data at the end of 2025. This is also corrected in the manuscript.
5	The manuscript classifies MRSA as: Hospital-associated (85.2%). Yet 88.5% are from outpatients. This is contradictory. Clearly define how HA-MRSA vs CA-MRSA was determined. If classification is based on facility rather than epidemiological criteria, clarify this limitation.	Here, the classification was dependent on to show that this data provides some clue and creates awareness for clinicians and the community. We have removed the phrases "HA-associated and CA-associated". We explained only as the isolates were from hospitals when compared with other facilities like health centers and clinics. In addition, we

		described also whether the isolates were from OPD when compared to IPDs.
6	The analysis is purely descriptive. No statistical tests are reported: No comparison between groups (e.g., age, sex). No trend analysis (despite a 5-year dataset). Include: Chi-square or logistic regression (e.g., MRSA vs MSSA predictors), trend analysis (e.g., regression) and confidence intervals consistently for key outcomes	Because we used the available data retrospectively, the registration book did not contain other factors
7	Some interpretations are speculative without supporting data: "Increased MRSA burden may stem from poor stewardship or infection control". These claims are reasonable but not directly measured. Rephrase to reflect a hypothesis rather than conclusion. Clearly distinguish data-driven findings vs. assumptions.	We have now removed such speculations as there were no supporting data.
8	The study uses disk diffusion only, which is acceptable. However, no mention of: D-test for inducible clindamycin resistance. MIC confirmation for key drugs (e.g., vancomycin). Mention whether D-test was performed (important for clindamycin interpretation). Clarify absence of MIC testing as a limitation	Additional tests were not done due resource constraints and this is one of the limitations. All corrected in the manuscript.
9	Important anti-MRSA agents are missing: Vancomycin, Linezolid, Teicoplanin. Explain why these were not tested (resource limitation?) Discuss implications for treatment recommendations	These antibiotic discs were not available at the time due to resource limitations. If these antibiotics are recommended as determined by physician, referring or searching for the antibiotics from other sources was mandatory.
10	MDR definition is appropriate. However: MARI calculation lacks clarity: were all isolates tested against the same number of antibiotics? Clarify denominator consistency. Explain variation (e.g., nitrofurantoin only	Here, all the isolates were tested against the same antibiotics except isolates from urine. In addition to other antibiotics, urine isolates were tested against Nitrofurantoin.
11	Some inconsistencies: Nitrofurantoin totals do not match full sample size. Percentages occasionally unclear (denominator not specified). Ensure all percentages clearly state denominators and separate urine-only analysis where appropriate.	As per current CLSI guidelines, nitrofurantoin should be tested for urine isolates in addition to other antibiotics. Now we have removed nitrofurantoin to standardize the denominator
12	Several sentences are overly long, grammatically inconsistent, and repetitive. Examples: "Patient demographic characteristics (age and sex), type of referring health facilities..." → incomplete sentence; "Clinical specimens... and antimicrobial susceptibility profiles." → fragment. Perform thorough language editing and consider professional proofreading	Corrected in the manuscript
13	Figure 1: Not clearly labeled (axes unclear) and MSSA vs MRSA trend needs better explanation	Removed

14	Tables: Formatting inconsistencies and some spacing and alignment issues	Corrected
15	Discussion: Generally good comparison with literature. However: too descriptive and needs more critical	Corrected
16	Resolve HA vs CA inconsistency and improve antimicrobial testing discussion	Corrected
17	Limitations: Important points mentioned, but missing: Lack of molecular typing, clinical outcome data, and antibiotic usage data.	Since the study was retrospective, molecular testing and determinants were not included. In addition, due to supply constraints, some antibiotics were not tested
18	The conclusion also requires revision	

2. **Reviewer 2**

SN	Reviewer comments	Authors' response
1	Identification of MRSA relied solely on the phenotypic cefoxitin disk diffusion method. No molecular assays (e.g., mecA gene PCR and PVL toxin gene detection) were performed. Consequently, the study cannot distinguish healthcare-associated (HA-MRSA) from community-associated (CA-MRSA) strains, nor can it elucidate the transmission mechanisms of resistance genes. Although the authors acknowledge this limitation in the manuscript, mere acknowledgment is insufficient. At a minimum, detection of one key resistance gene (e.g., mecA) should be performed; otherwise, the manuscript lacks convincing evidence.	Molecular analysis was not performed because of absence of the method. In addition, we were unable to differentiate HA-MRSA and CA-MRSA by this study. We only stated whether the MRSA isolates were from outpatient department or from inpatient departments.
2	The cross-sectional design only allows description of associations; it cannot determine the causal direction (e.g., whether hospitalization leads to MRSA infection or infection leads to hospitalization).	Due to its retrospective nature, we cannot determine casual direction with this study. However, the information regarding MRSA and its antimicrobial resistance pattern is believed to provide valuable concepts nationally, regionally and locally.
3	Key variables such as previous antibiotic use, hospitalization history, underlying diseases, and medical procedures were not collected. Therefore, a multivariate analysis to identify independent risk factors for MRSA infection could not be performed	It is just a retrospective data analysis and hence associated factors were not collected.
4	Although the manuscript reports that multiple specimen types were collected, 67% of MRSA isolates originated from middle ear discharge, while blood, urine, pus, sputum, and other common infection sites together accounted for only 33%. This distribution markedly differs from global patterns where MRSA is mainly isolated from wounds, the respiratory tract, and blood. The bias may reflect preferential submission of specimens from otitis media patients or a high local prevalence of otitis media. Thus, the results cannot be extrapolated to the overall epidemiology of MRSA in this region; interpretations should be restricted to "otitis-media-associated MRSA" or should explicitly note the referral bias	In the current study area, middle ear discharge is the most common clinical specimen referred for testing than other specimens. Number of MRSA isolates was therefore raised in this specimen.
5	First-line and second-line agents for treating MRSA infections (e.g., vancomycin, linezolid, daptomycin) were not tested. The study relied on agents such as gentamicin (which is relatively toxic and not the preferred	In the area, linezolid is not commonly available unless referred to other areas. Vancomycin and Daptomycin testing is by MIC which is not available at the microbiology laboratory.

	choice for MRSA), greatly reducing the clinical value of the findings for guiding therapy.	
6	The presentation of the five-year MRSA trend (Figure 1) is inadequate. Currently, only absolute case numbers are described without graphical support or statistical analysis. The authors should provide a bar chart (or line graph) showing both annual absolute counts and, more importantly, the MRSA detection rate (MRSA / total S. aureus isolates × 100%) for each year. A statistical test for trend, such as the Cochran-Armitage test for trend or logistic regression with year as a continuous variable, should be performed and the corresponding P value reported. This would allow readers to assess whether the observed increase is statistically significant or merely due to random variation or changes in sample submission volumes over time.	Figure 1 replaced by table which is appropriate in this context. All corrected in the manuscript.

Re: Spectrum00856-26R1 (**Mapping the Antimicrobial Susceptibility of Methicillin-Resistant *Staphylococcus aureus* in Western Ethiopia: A retrospective cross-sectional analysis**)

Dear Mr. Endalu Tesfaye Guteta:

Any pertinent data should be made available; data upon request from author is not consistent with ASM data policy.

Your manuscript has been accepted, and I am forwarding it to the ASM production staff for publication. Your paper will first be checked to make sure all elements meet the technical requirements. ASM staff will contact you if anything needs to be revised before copyediting and production can begin. Otherwise, you will be notified when your proofs are ready to be viewed.

Data Availability: ASM policy requires that data be available to the public upon online posting of the article, so please verify all links to sequence records, if present, and make sure that each number retrieves the full record of the data. If a new accession number is not linked or a link is broken, provide production staff with the correct URL for the record. If the accession numbers for new data are not publicly accessible before the expected online posting of the article, publication may be delayed; please contact ASM production staff immediately with the expected release date.

Publication Fees: For information on publication fees and which article types have charges, please visit our website. We have partnered with Copyright Clearance Center (CCC) to collect author charges. If fees apply to your paper, you will receive a message from no-reply@copyright.com with further instructions. For questions related to paying charges through RightsLink, please contact CCC at ASM_Support@copyright.com or toll free at +1-877-622-5543. CCC makes every attempt to respond to all emails within 24 hours.

ASM Membership: Corresponding authors may join or renew ASM membership to obtain discounts on publication fees. Need to upgrade your membership level? Please contact Customer Service at Service@asmusa.org.

Embargo Policy: A press release may be issued as soon as the manuscript is posted on the Spectrum Latest Articles webpage. The corresponding author will receive an email with the subject line "ASM Journals Author Services Notification" when the article is available online.

The ASM Journals program strives for constant improvement in our submission and publication process. Please tell us how we can improve your experience by taking this quick Author Survey.

Thank you for submitting your paper to Spectrum.

Sincerely,
Deena Altman
Editor
Microbiology Spectrum
